# The Effect of COVID-19 Vaccine on Women's Reproductive Health: A Cross-Sectional Study

**DOI:** 10.7759/cureus.40076

**Published:** 2023-06-07

**Authors:** Razaz Wali, Hayat Alhindi, Arwa Saber, Khowlah Algethami, Reem Alhumaidah

**Affiliations:** 1 Family Medicine, King Abdulaziz Medical City, National Guard Hospital, Jeddah, SAU; 2 Family Medicine, King Saud Bin Abdulaziz University for Health Sciences College of Medicine, Jeddah, SAU; 3 Family Medicine, King Abdullah International Medical Research Center, Jeddah, SAU; 4 Medicine, King Saud Bin Abdulaziz University for Health Sciences College of Medicine, Jeddah, SAU

**Keywords:** covid-19 vaccine, primary healthcare center, decreased libido, miscarriage, covid-19 in pregnancy, menstrual cycle irregularity, breastmilk, saudi arabia, effect, reproductive health

## Abstract

Introduction: The coronavirus disease 2019 (COVID-19) vaccine was developed to stimulate acquired immunity against severe acute respiratory syndrome coronavirus 2 (SARS-CoV-2). Symptoms of reproductive health abnormalities have been reported following the administration of the adenovirus and mRNA-containing vaccine. Such complaints included irregular menstrual cycles, miscarriages, changes in sexual interest, vaginal bleeding, and decreased milk supply in breastfeeding mothers. This study aimed to explore the effect of the COVID-19 vaccine on the reproductive health of women attending five primary healthcare centers in the western region of Saudi Arabia.

Methods: A cross-sectional study was conducted with 300 women between 15 and 50 years. Five primary healthcare centers were included from May to September 2022. Non-probability convenient sampling technique was used; data were collected via a self-administered questionnaire from women who received any number or type of COVID-19 vaccine. Data were analyzed using Statistical Package for the Social Sciences (SPSS) version 22 (IBM SPSS Statistics, Armonk, NY, USA).

Results: Of those who responded to the questionnaire (297 participants), 74% were married, and 52% had 1-3 children. Of the pregnant women, only 4% lost their pregnancy. In addition, of the breastfeeding mothers, 10% noticed a decrease in milk production after the vaccination. The effect of the vaccination status on decreased libido was 11%. A small proportion (18%) of the participants reported worsening dietary habits after the vaccine. Less than half of the participants (44%) reported a change in the length and amount of the menstrual cycle, and 29% worsened premenstrual syndrome (PMS). There was no significant association between the type and the number of doses on the rate of miscarriage (p=0.47), breastmilk production (p=0.47), libido (p=0.11), health diet habits (p=0.15), monthly cycle (p=0.570), heavy menses (p=0.999), and PMS symptoms in the study participants.

Conclusion: COVID-19 vaccination remains necessary to prevent severe infection and is safe for females of reproductive age, whether trying to get pregnant or lactating, and has no significant effect on the menstrual cycle. This research can be used as a basis when deciding on vaccines in case of future pandemics and remove misinformation and doubts regarding the vaccines that should be adequately addressed.

## Introduction

The coronavirus disease 2019 (COVID-19) vaccine was developed to provide acquired immunity against severe acute respiratory syndrome coronavirus 2 (SARS-CoV-2). The COVID-19 vaccine was first introduced to the world following the pandemic declaration in March 2020. Vaccines were developed during the COVID-19 pandemic to prevent virus complications. According to the Saudi Center for Disease Control and Prevention, three vaccines are currently approved to be administered in the Kingdom of Saudi Arabia [1]. Two were developed to stimulate an RNA immune response, including Pfizer-BioNTech and Moderna. The Oxford-AstraZeneca vaccine uses a non-replicating adenovirus shell containing DNA that encodes a SARS-CoV-2 protein to stimulate an immune response [2,3]. Frequent side effects of the vaccine include muscle pain, fever, arm soreness, headaches, and fatigue [4]. Although there was no listing of menstrual cycle changes and related reproductive health symptoms, there has been some speculation about the effects of the vaccine on women's reproductive health. Primary health workers have received multiple complaints in daily practice from premenopausal women soon after receiving the vaccine. Such complaints included irregular menstrual cycles, miscarriages, changes in sexual interest, and vaginal bleeding. Also, breastfeeding mothers reported decreased milk production [4,5].

Symptoms of reproductive health abnormalities were reported following the adenovirus and mRNA-containing vaccines. The possible effect of the immunological response after administering the COVID-19 vaccine requires acknowledgment. The theory suggests that the reported reproductive symptoms may result from an immune activation leading to hormonal changes [5].

As we continually learn about COVID-19 vaccines, new questions arise regarding their effect on reproductive health. However, limited data links COVID-19 vaccines and menstrual cycle changes [6]. A retrospective observational case series analysis in Ghana reported three cases of abnormal uterine bleeding post-COVID-19 vaccination [7]. Similarly, in March 2021, a sample of vaccinated women in the United Kingdom reported that vaccination for COVID-19 caused menstrual disturbances in 20% of women [6]. A retrospective study with 1,273 participants at Imperial College London failed to discover a significant association to support that COVID-19 vaccination is linked to menstrual changes [5].

Millions of women have become pregnant, given birth, and initiated breastfeeding during the COVID-19 pandemic. However, the persistent exclusion of pregnant and lactating women from the COVID-19 antiviral and vaccine trials created a knowledge gap regarding the outcome of pregnancy and breastfeeding [8].

In a prospective cohort study at two tertiary care centers, 131 reproductive-aged vaccine recipients (84 pregnant, 31 lactating, and 16 non-pregnant women) reported that mRNA-based COVID-19 vaccines generated robust humoral immunity in pregnant and breastfeeding females, with immunogenicity and reactogenicity being similar to non-pregnant women [9]. Breastfeeding women in the United States who received the mRNA vaccine were enrolled in the Mommy's Milk Human Milk Research Biorepository at the University of California, San Diego. A small number reported a reduction in milk supply and a change in the color of milk. However, milk production returned to normal within 72 hours [10].

In terms of fertility concerns, studies in the region reported several in vitro fertilization (IVF) parameters, such as the number of oocytes, the rate of fertilization, and the ratio of top-quality embryos (TQEs) per fertile oocyte in women who received the mRNA vaccines similar to unvaccinated women [11]. A Jordanian cross-sectional study mentioned self-reported incidences of irregular menses and decreased libido [12]. Locally, a Saudi Arabian study evaluating early solicited adverse events following the BNT162b2 mRNA vaccination also reported delayed menstruation as one of the responses [13]. Other than that, there is a lack of research in this area, which we intend to explore.

In conclusion, although COVID-19 vaccines have life-saving benefits, it is vital to explore the presence of any effects on reproductive health, including menstrual changes, libido, and conception. Our study investigated such effects, factors, and possible associations to reduce the knowledge gap and inform healthcare providers and the public.

## Materials and methods

This study was cross-sectional, with women who attended the maternity and family medicine clinic in five primary healthcare centers. The centers included Iskan Clinic, Specialized Polyclinic, Bahraa, staff health, and Shareae primary healthcare centers. All are only satellite clinics of King Abdulaziz Medical City (KAMC).

The total number of women who attended the clinics from March to June 2022 was more than 500, and the required sample size was 300, calculated using Raosoft software (http://www.raosoft.com/samplesize.html) at a 95% confidence interval (CI) level with a response distribution of 50% and a margin of error of ±5.

The sample was obtained using a non-probability convenient sampling technique to select study participants from the list of patients booked at the maternity and family medicine clinics. Data was collected using an author-developed questionnaire. Most (80%) of the patients used a questionnaire as a Google Forms (Google, Inc., Mountain View, CA, USA) collected by direct interview. The remaining 20% completed a self-administered questionnaire using Google Forms sent via AirDrop. Three questionnaires were excluded because they did not meet the inclusion criteria. The consent form is on the first page, which was mandatory to complete before they could proceed to the next section. They could open the link, respond to the questions, and submit after the final response. The inclusion criteria were women between the age of 15 years and 50 years who received one or more COVID-19 vaccines of any type.

The questionnaire consisted of seven parts: (1) demographic data, including age, height, weight, residency, marital status, occupation, and number of children; (2) data related to general health status, including any chronic disease, medication list, thyroid disease, exposure to COVID-19 infection, smoking, diet, and exercise; (3) vaccine dose and date, including the number of doses and the type of vaccine; (4) information about the menstrual period, including the record of cycles using a diary/smartphone application or other methods, regular or irregular, days of bleeding on average during the period, heavy bleeding or not, painful period or not, missed period, and any change in the premenstrual symptoms; and (5-7) obstetrical and gynecological data during COVID-19 and after getting coronavirus vaccine doses.

For the reliability of the questionnaire, the consistency in responding over time and consistency in responding to similar questions were addressed. The questionnaire was sent to experts in the field for content validity. In contrast, face validity was ensured by sending the questions to colleagues who are experts in writing and validating questionnaires.

Ethical approval for the study was obtained from the Institutional Review Board (IRB) of King Abdullah International Medical Research Center (KAIMRC) with IRB approval number IRB/0389/22. Ethical principles were maintained throughout the research process.

Data were entered into a personal computer using Microsoft Excel software (Microsoft Corp., Redmond, WA, USA). Data were accessed only by the researchers, and it was analyzed using Statistical Package for the Social Sciences (SPSS) version 22 (IBM SPSS Statistics, Armonk, NY, USA). Continuous data are presented as means and standard deviations and categorical variables as frequencies and percentages. A p-value of <0.05 was considered significant. The Chi-square test was used for inferential statistics to compare categorical variables and describe any significant association between the item of reproductive health and the number of vaccines received.

## Results

Demographic and general health status characteristics

A total of 297 vaccinated females of reproductive age, between the ages of 15 and 50, completed the questionnaire with a response rate of 100%. The highest proportion (n=143, 48%) of the participants was from 21 to 32 years. The majority (74%, n=219) of the participants were married women (Table 1). A small proportion (n=64, 22%) had a chronic disease. Asthma (6%) and hyperthyroidism (5%) were the most frequent (Table 1).

**Table 1:** Participants' demographic characteristics (N=297) BMI: body mass index

Demographic characteristics		Number	%
Age	15-20 years	15	5
	21-26 years	72	24
	27-32 years	71	24
	33-38 years	66	22
	39-44 years	43	14
	45-50 years	24	8
	>50 years	6	2
Marital status	Single	70	23
	Married	219	74
	Divorced/widowed	8	3
How many children (n=222)	None	42	19
	1-3	115	52
	4-6	54	24
	>6	11	5
Any chronic disease		64	22
Type of chronic disease (n=64)	Diabetes	10	3
	Hypertension	11	4
	Asthma	19	6
	Hypothyroidism	16	5
	Any other disease	21	7
BMI (n=289) (kg/m^2^)		25.85 ± 5.62	

Vaccination status

Of those who responded to this question, the majority (n=279) received the first dose of the COVID-19 vaccine, of which 74% received Pfizer, 25% received Oxford-AstraZeneca, and 1% received Moderna. On the other hand, of the 295 who received the second dose, 76% received Pfizer, 19% received Oxford-AstraZeneca, and 5% received Moderna. Most (n=187, 63%) of the 297 received the third dose, mostly Pfizer (n=151, 81%).

COVID-19 vaccine and conception/miscarriage

There were 219 (74%) married respondents (Table 1), and 47 (21%) were pregnant. Of the remaining 172, 38 (22%) were trying to conceive during this period, and 20 (53%) reported being successful, with three (8%) reporting a miscarriage during their pregnancy (Table 2).

**Table 2:** Impact of COVID-19 vaccination on reproductive women's health ^a^Fisher's exact test, ^b^Chi-square test COVID-19: coronavirus disease 2019

Item		Total	Number of COVID-19 vaccine doses received				p-value
			Two		Three		
			Number	%	Number	%	
If you are breastfeeding, did you notice any changes?	Decreased milk supply	2	0	0	2	25	0.47^a^
	Milk supply unchanged	14	8	100	6	75	
Did you notice any change in libido after receiving the COVID-19 vaccine?	Increased libido	1	1	2	0	0	0.11^a^
	Decreased libido	34	9	14	25	25	
	Unchanged	129	53	84	76	75	
After receiving the vaccine, did you suffer from any miscarriages?	Yes, after the first dose	6	3	3	3	2	0.47^a^
	Yes, after the second dose	2	1	1	1	1	
	Yes, after the third dose	1	1	1	0	0	
	No	250	92	95	158	98	
Did you notice any changes in your diet during the pandemic?	Overall, diet is better	61	29	27	32	17	0.15^b^
	Overall, diet is worse	53	19	18	34	18	
	Unchanged	179	60	56	119	64	

The impact of vaccination on breastfeeding, libido, and dietary habits

Table 2 displays the effect of vaccination on 16 breastfeeding women: 14 (87.5%) expressed an unchanged breast milk supply, and a decreased supply was noticed in two (12.5%) of the lactating mothers. There was no significant relation comparing the type and the number of doses and breastmilk production in breastfeeding mothers (p=0.47).

In addition, of the sexually active women (n=164), the effect of the COVID-19 vaccine on libido was the same in 129 (78%) women, with reduced sexuality reported by 34 (20.7%) respondents. There was no significant association between the COVID-19 vaccine and a change in libido (p=0.11).

The majority (n=179, 61%) of the women reported no change in their dietary habits after taking the vaccine; 61 (21%) indicated that their diet was better, and 53 (18%) stated that it was worse. No association was found between vaccination and change in diet (p=0.15).

Menstrual cycle and vaccination

A small proportion (20%, n=60) of the sample reported irregular periods before the COVID-19 vaccine. The remaining 80% (n=237) reported no evidence of menstrual alteration. No statistically significant differences were observed regarding the regularity of the menstrual cycle in individuals who received the first, second, and third COVID-19 vaccine, at 297 (79.7%), 295 (80%), and 187 (78%), respectively, in their monthly menstrual flow (p=0.57, p=0.07, and p=0.23, respectively) (Table 3).

**Table 3:** Impact of COVID-19 vaccination on the menstrual cycle COVID-19: coronavirus disease 2019

Number of doses	Number	Regular menstrual cycle after receiving the vaccine dose				p-value
		Yes		No		
		Number	%	Number	%	
First dose	297	237	79.7	60	20.2	0.57
Second dose	295	236	80	59	20	0.07
Third dose	187	146	78	41	21.9	0.23

After receiving the COVID-19 vaccine, 22 (9.69%) of 227 first-dose recipients and 33 (14.6%) of 225 second-dose recipients expressed heavy menses (p=0.001), and 20 (16.39%) of 122 reported the same issue after the third dose. There were no statistically significant differences in the heaviness of the menstrual period (p=0.17, p=0.06, and p>0.999, respectively) (Table 4).

**Table 4:** Impact of COVID-19 vaccination on menstrual flow COVID-19: coronavirus disease 2019

Number of doses	Number	Heavy periods after receiving the vaccine dose				p-value
		Yes		No		
		Number	%	Number	%	
First dose	227	22	9.69	205	90.3	0.17
Second dose	225	33	14.6	192	85.3	0.06
Third dose	122	20	16.39	102	83.6	>0.999

When comparing the groups who received one, two, or three doses of the vaccine, 35 (21%) stated they had "painful periods" after the first dose, 47 (28%) after the second dose, and 28 (17%) after the third dose, which was not clinically significant (p=0.55, p=0.50, and p=0.71, respectively) (Table 5). All participants were asked about missing menstrual cycles. Following COVID-19 vaccinations, there was no significant association between the first, second, and third doses and missing the menstrual cycle (p>0.999, p>0.999, and p=0.48, respectively) (Table 6). Of women with premenstrual syndrome (PMS), 69% declared no change in their symptoms after receiving COVID-19 immunization, 29% had worse symptoms, and 3% had better symptoms.

**Table 5:** Impact of COVID-19 vaccination on menstrual pain COVID-19: coronavirus disease 2019

Number of doses	Number	Painful periods after receiving the COVID-19 vaccine dose				p-value
		Yes		No		
		Number	%	Number	%	
First dose	167	35	20.9	132	79	0.55
Second dose	166	47	28.3	119	71.68	0.50
Third dose	89	28	31.4	61	68.5	0.71

**Table 6:** Impact of COVID-19 vaccination on missing menstrual cycle COVID-19: coronavirus disease 2019

Number of doses	Number	Miss any period after the dose						p-value
		Yes, always		Yes, often		No		
		Number	%	Number	%	Number	%	
First dose	233	3	1.28	15	6.43	215	92.2	>0.999
Second dose	232	7	3.01	24	10.34	201	86.6	>0.999
Third dose	131	4	3.05	13	9.92	114	87	0.48

## Discussion

The COVID-19 pandemic, a major global coronavirus outbreak in 2020 caused by the SARS-CoV-2 virus, raised many concerns regarding its effect on the female reproductive system. Although the COVID-19 vaccine was established to provide acquired immunity against COVID-19, cases of menstrual cycle changes and related reproductive health symptoms, miscarriages, changes in sexual interest, vaginal bleeding, and decreased milk production in breastfeeding mothers were reported [3,4]. This study aimed to evaluate the presence of reproductive health changes following the administration of the COVID-19 vaccine in women of reproductive age in the primary healthcare settings in the National Guard Health Affairs, Jeddah.

This research included 297 vaccinated premenopausal reproductive-aged females in the regular healthy group, excluding hormonal factors due to obesity or anorexia that can affect the study's results regarding reproductive health [2]. The type of vaccine administered was reported in three different doses. Most participants received the first dose of Pfizer, and the main reason was the availability of the vaccine in the centers. However, about a third of the participants received the third dose, which may have been due to misconception and lack of awareness.

COVID-19 vaccine effect on fertility

Fertility problems have been a concern in the community following the COVID-19 vaccine. However, in this study, approximately half of the participants who were trying to conceive succeeded, and a small percentage experienced a miscarriage, indicating that the vaccine is safe and is not associated with pregnancy loss or infertility. More than half had children before the vaccine, indicating their fertility. Similarly, a review stated that males and females had no fertility issues or adverse pregnancy outcomes after the vaccination. The benefits of the antibodies transferred through the placenta from the maternal side are more beneficial and outweigh any potential risks [14]. In the case of the rapid spread of COVID-19, it is more helpful to be vaccinated instead of taking the risk of severe adverse symptoms due to SARS-CoV-2 virus infection. A systematic review indicated no scientific proof or association between COVID-19 vaccines and fertility impairment in men or women [15].

In addition, no significant association was found between the effect of the COVID-19 vaccine and libido. A study illustrated that the COVID-19 vaccines are safe, and no evidence suggests any negative impact on fertility or sexual health, instead of the associated long-term consequences likely to develop due to COVID-19 itself [16].

COVID-19 vaccine effect on breastfeeding

There was a concern from lactating females whether the COVID-19 vaccine would be transferred to their infant. No significant relationship was found in the breastfeeding candidates in this study by comparing the type and the number of doses in the reproductive health variables. The vaccine in lactating mothers is considered safe. Pfizer and Moderna reported a temporary reduction in milk supply in a cohort study involving 180 women who received the COVID-19 vaccines. With the Pfizer vaccine, 7.3% and 8% showed a reduction after the first and second dose, respectively, and with the Moderna vaccine, it was 8% and 23.4%, respectively. The main difference between the two types of vaccines was statistically significant after the second dose. However, the milk supply returned to normal within three days in all cases [17].

Some women reported an increase in milk supply after each dose, and five women reported a blue-green change in milk color after the first dose of the vaccine. The changes in lactating mothers were temporary and did not affect breastfeeding or the infant [17]. Another study found that none of the breastfed infants of 50 women who received the COVID-19 vaccine during pregnancy or lactation had any serious adverse effects. Only a few reported minor sleep changes and gastrointestinal symptoms after the first dose of the vaccine. The majority (88%) of the women reported no infant side effects and none after the second dose [18].

COVID-19 vaccine effect on the menstrual cycle

Changes in the menstrual cycle were reported in the literature following vaccination. Although, in the current research, most participants had no evidence of menstrual alteration, some indicated an increase in menstrual flow. A Saudi Arabian study evaluated the impact of the COVID-19 vaccine on all features of the menstrual cycle, including cycle length, amount of bleeding, and pain. A total of 673 women were included, and changes in the menstrual cycle after both COVID-19 vaccine doses were observed in 46.7%, and more menstrual pain was noticed in 22.9% (p<0.001). The current research showed that in women with PMS, 69% reported no change in their symptoms after receiving the COVID-19 immunization, 29% reported worsening symptoms, and 3% reported improved symptoms [19]. A study reported that 78% of the participants that experienced menstrual cycle changes after vaccination were older and smokers compared with women who did not note any differences. The most predominant menstrual changes were more menstrual bleeding (43%), more menstrual pain (41%), delayed menstruation (38%), fewer days of menstrual bleeding (34.5%), and a shorter cycle length (32%). More menstrual cycle changes after the COVID-19 vaccines were observed in patients with chronic conditions, such as PMS.

## Conclusions

In conclusion, the COVID-19 vaccination is necessary to prevent severe infection and severe COVID-19 complications. This study promotes vaccine utilization as there is no statistically significant association between COVID-19 vaccination and breastfeeding problems, pregnancy loss or infertility, and menstrual cycle disorders. This research adds to the literature on the effect of the COVID-19 vaccine on women's reproductive health, which aligns with other studies in the literature.
